# Self-esteem in Moroccan women: comparison between different age groups and influencing factors

**DOI:** 10.1192/j.eurpsy.2022.2239

**Published:** 2022-09-01

**Authors:** A.K. Rhaouti, A. Khallouk, S. Belbachir, A. Ouanass, O. Belakbir

**Affiliations:** 1 Hôpital Ar-Razi, Men’s Unit B, Sale, Morocco; 2 University Psychiatric Hospital Ar-razi, university hospital center, SALE, Morocco, University Of Mohammed V, Rabat,, salé, Morocco; 3 Hôpital psychiatrique ARRAZI de Salé, Psychiatrie, Salé, Morocco; 4 Psychiatric University Hospital Ar-razi, Men’s Unit B, salé, Morocco

**Keywords:** self-esteem, women

## Abstract

**Introduction:**

This study revolves around self-esteem which is defined as a basic human characteristic related to self-awareness, emotions, cognitions, behavior, lifestyle, general health and socio-economic factors. This fundamental data of the personality is revealed from one person to another as well as from one period to another. Many studies point out that advancing adults do not necessarily imply a decline in sense of self-worth, although skill losses are very real in many areas of psychological activity. Therefore, it seemed interesting to us to further explore this point in Moroccan women.

**Objectives:**

Evaluate and compare self-esteem among different age groups of Moroccan women. Identify the different influencing factors.

**Methods:**

This is a descriptive cross-sectional study using a questionnaire based on tow parts, the first based Rosenberg scale, and the second part to identify the presence of certain factors influencing self-esteem.

**Results:**

Our researches have shown a similar results to those of some previous studies. Indeed, we found out that women experience a significant rise of self-esteem simultaneously with the increase of age. Yet, this self-esteem starts to decline in middle-aged women. Several factors can affect it; we can note on the top, the impact of relationships, education and physical health.

**Conclusions:**

This research contributes to our understanding of Moroccan women’s self-esteem and to the identification of factors that influence it.Self-esteem is a core identity issue, essential to personal validation and our ability to experience joy. Previous researchs also suggests that self-esteem might influence economic welfare and physical health.

**Disclosure:**

No significant relationships.

